# Munchausen Syndrome Presented as Guillain-Barré Syndrome: A Case Report and Literature Review

**DOI:** 10.7759/cureus.77057

**Published:** 2025-01-07

**Authors:** Luís Paulino Ferreira, Janice Alves, Joana Marta, Gonçalo V Bonifácio, Andre Militão

**Affiliations:** 1 Department of Psychiatry and Mental Health, Unidade Local de Saúde da Arrábida, Setúbal, PRT; 2 Department of Neurology, Unidade Local de Saúde da Arrábida, Setúbal, PRT

**Keywords:** case report, diagnostic challenges, factitious disorder, munchausen syndrome, neurology and psychiatric disorders

## Abstract

Munchausen syndrome (MS), a complex form of factitious disorder (FD), presents significant diagnostic and management challenges in emergency and hospital settings. Patients deliberately fabricate or induce symptoms to gain medical attention, often leading to unnecessary interventions, resource misallocation, and iatrogenic harm. This study highlights the diagnostic complexity and the need for multidisciplinary management of Munchausen syndrome through a detailed case report and literature review. A 30-year-old woman presented with neurological symptoms mimicking Guillain-Barré syndrome (GBS), including quadriplegia and sensory deficits. Inconsistencies during physical examination and falsified imaging reports prompted further investigation, uncovering a history of fabricated symptoms and pathological lying. The psychiatric evaluation confirmed the diagnosis of Munchausen syndrome. Differentiating Munchausen syndrome from malingering, conversion disorder, and somatic symptom disorders requires meticulous evaluation and interdepartmental collaboration. Unlike malingering, Munchausen syndrome lacks external incentives, with psychological factors such as trauma and personality disorders playing a central role. Early recognition is essential to prevent unnecessary procedures, reduce costs, and avoid prolonged hospitalizations. This case underscores the need for clinical vigilance and a systematic approach to diagnosis. A multidisciplinary strategy involving psychiatry and other specialties is vital for effective management and improved patient outcomes.

## Introduction

Factitious disorders (FD) present a unique challenge to healthcare providers, especially in the emergency department (ED), given the need to effectively triage based on illness severity and ensure swift diagnosis, crucial for effective care. Conditions involving intentional exaggeration, fabrication, simulation, aggravation, or self-induction of symptoms pose significant challenges for ED personnel, being both diagnostically complex and mentally demanding to manage [1].

The term "Munchausen syndrome" (MS), coined in 1951 by Dr. Richard Alan John Asher, was inspired by Baron Karl Friedrich von Münchhausen (1720-1797), an 18th-century figure famous for his fantastical and exaggerated tales of adventure [2,3]. Dr. Asher applied this name to a psychiatric disorder involving patients who fabricated or exaggerated symptoms, often presenting with neurological, hematological, or gastrointestinal complaints. Individuals with MS frequently have severe co-occurring personality disorders, though the exact relationship between them remains unclear. By linking the patients' fabricated symptoms to Baron's embellished stories, Dr. Asher established a term that highlights the diagnostic complexity and historical resonance of this condition throughout the medical literature [3].

The Diagnostic and Statistical Manual of Mental Disorders, Fifth Edition (DSM-5) categorizes factitious disorders into two types: those imposed on oneself and those imposed on others (formerly referred to as "factitious disorder by proxy"). These conditions are characterized by the intentional fabrication or exaggeration of physical and/or psychological symptoms, as well as the deliberate induction of illness or injury, often accompanied by evidence of deception [4,5].

Guillain-Barré syndrome (GBS) is an acute, immune-mediated inflammatory polyradiculoneuropathy with several subtypes. It is characterized by rapidly progressive, bilateral muscle weakness, typically ascending and beginning in the distal lower limbs, accompanied by sensory disturbances, ataxia, and autonomic dysfunction. Most patients exhibit reduced or absent deep tendon reflexes. Symptoms usually progress over 2-4 weeks, with 20%-30% of patients requiring mechanical ventilation due to respiratory failure. GBS is often preceded by an infection (bacterial or viral) or, less commonly, by vaccination. Prognosis varies widely, ranging from full recovery within weeks to death, with approximately 15%-20% of patients experiencing residual deficits. Symptoms typically peak between days 10 and 14, followed by recovery over weeks to months, which may be slower in cases involving axonal damage. Relapses are rare, as the disease typically follows a monophasic course [6].

This review aims to provide a concise overview of MS and its variants for students and healthcare professionals. Factitious disorders are often underrecognized or misdiagnosed but can be present in emergency rooms (ER), clinical units, and other healthcare settings, posing significant diagnostic and management challenges. To improve the understanding of these overlooked disorders, this article begins with a case report, followed by a focused literature review.

## Case presentation

Emergency department presentation

A 30-year-old woman, a medical doctor, single but recently in a relationship with a healthcare provider, was brought to the ER with an altered state of awareness. Her symptoms began 24 hours ago with a sudden onset of tingling, which subsequently progressed to paraparesis. Her past medical history, as self-reported, is significant for several conditions. Since the age of two, she has experienced monthly episodes of high fever (39°C-41°C) lasting 1-2 weeks before spontaneous remission. She was diagnosed with tumor necrosis factor (TNF) receptor-associated periodic syndrome (TRAPS) and adenosine deaminase 2 deficiency (DADA2), according to the patient, at the age of 13, confirmed by genetic testing. She reported starting colchicine (2 mg/day) shortly after the diagnosis. At the age of 21, she was reportedly diagnosed with immunoglobulin A (IgA) nephropathy based on routine blood work indicating renal failure, although no pathological confirmation was available. Additionally, she reported being diagnosed with neurocysticercosis after a trip to Thailand eight years prior. According to the patient, the symptoms at the time included bilateral tonic-clonic seizures, headaches, nausea, vomiting, and sensory deficits. She stated that cranial computed tomography (CT) and lumbar puncture (LP) were performed; however, the results are not available, and she did not provide any documented record of the diagnosis or treatments received. Similarly, she did not present any records to substantiate an appendectomy at the age of 29. Her history also includes severe allergic reactions to fish, seafood, citrus, nonsteroidal anti-inflammatory drugs (NSAIDs), tramadol, and corticosteroids (except dexamethasone). She takes colchicine at a dose of 2 mg/day (1 mg in the morning and 1 mg at night), with no contributory family history and no reported substance use.

On initial examination, her vital signs were stable (blood pressure, 130/71 mmHg; heart rate, 66 beats per minute {bpm}; respiratory rate, 17 respirations per minute {rpm}; and oxygen saturation, 99%). She did not exhibit any respiratory difficulty, with no use of accessory muscles or intercostal retraction. The neurological assessment revealed that she was alert, oriented, and cooperative. Quadriplegia was observed with a Medical Research Council (MRC) muscle power score of 0/5 and generalized decreased pinprick sensation, while the cranial nerve examination was unremarkable. A slight reduction in cervical flexion strength was observed (MRC muscle power score of 4/5); however, the patient's limited cooperation during testing was noted.

**Figure 1:**
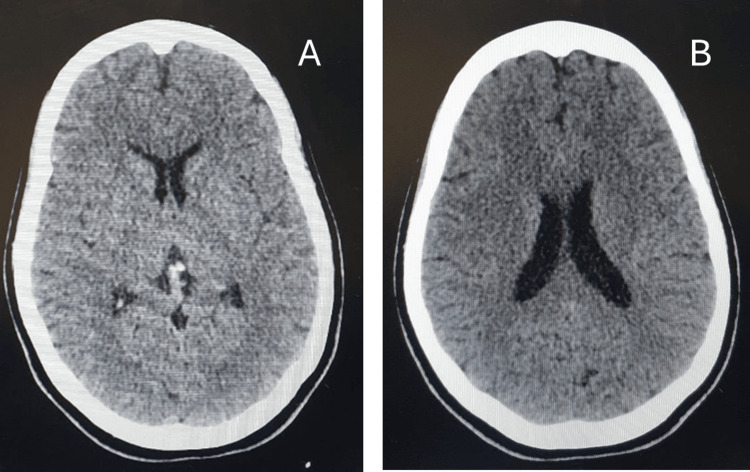
Cranial computed tomography (CT) (A) Non-contrast computed tomography (CT) of the brain showing an inferior axial section. No acute intracranial pathology or significant abnormalities were identified. (B) Non-contrast computed tomography (CT) of the brain demonstrating a superior axial section. No evidence of acute intracranial pathology or clinically relevant findings was identified

Initial investigations, including a cranial CT (Figure 1), electromyography (EMG) with nerve conduction study, and laboratory workups, including erythrocyte sedimentation rate (ESR), complete blood count (CBC), serum electrolytes, vitamin B12 levels, and renal, thyroid, and liver function tests, were all within normal limits, except for low a lactate dehydrogenase (LDH) level of 107 U/L (Table 1). The nerve conduction study was requested during the emergency evaluation; however, it could not be performed promptly due to long waiting times and limited resources. Based on the clinical presentation, GBS was suspected due to the findings of paraparesis progressing to quadriplegia with an MRC muscle power score of 0/5 and decreased sensation to pinprick. The patient was transferred to the Intermediate Medical Care Unit (IMCU) for further evaluation and the initiation of treatment.

**Table 1:** Laboratory test results aCL, anti-cardiolipin antibodies; ACPA, anti-citrullinated peptide antibodies; AHA, anti-histone antibodies; ALT, alanine aminotransferase; AMA, anti-mitochondrial antibodies; ANA, antinuclear antibodies; ANCA, antineutrophil cytoplasmic antibodies; Anti-β2GP1, anti-beta2-glycoprotein antibodies; Anti-dsDNA, anti-DNA antibodies; Anti-LC1, anti-LC1 antibodies; Anti-LKM1, anti-LKM1 antibodies; Anti-SLA, anti-SLA antibodies; ANuA, anti-nucleosome antibodies; AP, alkaline phosphatase; APCA, anti-parietal cell antibodies; APTT, activated partial thromboplastin time; ASMA, anti-smooth muscle antibodies; AST, aspartate aminotransferase; CRP, C-reactive protein; CSF, cerebrospinal fluid; D-B, direct bilirubin; ENAs, extractable nuclear antigens; GGT, gamma-glutamyl transferase; LDH, lactate dehydrogenase; PC, platelet count; PT, prothrombin time; RBC, red blood cells; RF, rheumatoid factor; T-B, total bilirubin; TP, total proteins; WBC, white blood cells; CBC, complete blood count; PR3, proteinase 3; MPO, myeloperoxidase; Ig, immunoglobulin

Test	Parameter	Result	Reference Range	Units
CBC	RBC	4.1	3.8-5.8	×10^6^/mL
	Hemoglobin	13.5	11.5-15	g/dL
	WBC	5.5	4.5-11.4	×10^3^/mL
	PC	162	150-350	×10^3^/mL
Biochemistry	PT	13.8	9.4-12.5	Seconds
	APTT	27.4	25.1-36-5	Seconds
	Albumin	3.4	3.5-5.0	g/dL
	TP	6.5	6.4-8.3	g/dL
	Glucose	107	70-105	mg/dL
	Urea	27.4	15-40	mg/dL
	Creatinine	0.71	0.6-1.1	mg/dL
	AST	14	5-34	U/L
	ALT	21	<55	U/L
	LDH	107	125-230	U/L
	GGT	9	9-36	U/L
	AP	57	40-150	U/L
	Sodium	137	136-146	mmol/L
	Potassium	3.6	3.5-5.1	mmol/L
	Chloride	108	98-107	mmol/L
	Calcium	8.8	8.4-10.2	mg/dL
	Phosphorus	3.7	2.7-4.5	mg/dL
	Magnesium	1.8	1.6-2.6	mg/dL
	T-B	0.59	<1.2	mg/dL
	D-B	0.14	<0.5	mg/dL
	CRP	0.10	<0.5	mg/dL
Protein Electrophoresis	TP	6.5	6.4-8.3	g/dL
	Albumin	56.8	55.8-66.1	%
	Alpha 1	3.7	2.9-4.9	%
	Alpha 2	7.9	7.1-11.8	%
	Beta 1	5.5	4.7-7.2	%
	Beta 2	3.3	3.2-6.5	%
	Gamma	22.9	11.1-18.8	%
Autoimmunity	RF	<8	<15	UI/mL
	ANA	Negative	-	-
	ENAs	Negative	<7	-
	Anti-dsDNA	8.4	<10	UI/mL
	ANCA			
	PR3	<0.2	<2	UI/mL
	MPO	<0.2	<3.5	UI/mL
	AMA	Negative	-	-
	ASMA	Negative	-	-
	Anti-LKM1	Negative	-	-
	Anti-SLA	Negative	-	-
	Anti-LC1	Negative	-	-
	APCA	3.6	<7	U/mL
	aCL			
	IgM	0.9	<10	U/mL
	IgG	2.3	<10	U/mL
	Anti-β2GP1			
	IgM	<2.9	<7	U/mL
	IgG	2.0	<7	U/mL
	AHA	Negative	-	-
	ANuA	Negative	-	-
	ACPA	1.8	<7	U/mL
CSF	Cytological Examination			
	Leukocytes	<5	-	mL
	Erythrocytes	-	-	-
	Chemical Examination			
	Glucose	68	40-70	mg/dL
	TP	33	15-40	mg/dL
	Bacteriological Examination	Negative	-	-

Intermediate medical care unit: Neurology consultation (day 2)

When further assessed in the IMCU, the patient reported a prior episode of lower limb sensory deficits, which resolved completely following corticosteroid treatment at another hospital (the type or dosage of the corticosteroids is unknown; she does not provide any written evidence of the treatment). She was also revealed to have undergone a cervical and brain magnetic resonance imaging (MRI) four months before, which, according to her, revealed multiple white matter lesions and was accompanied by a written report; however, she did not provide the images to confirm this information.

On clinical examination, she presented with quadriparesis, brisk reflexes in the lower limbs, absent radial reflexes, and normal bicipital reflexes. Superficial abdominal reflexes were absent, and there was decreased proprioceptive sensation in the upper limbs. Additionally, a left-sided peripheral facial palsy was observed.

Supplementary diagnostic investigations included a cervical CT scan, which revealed no abnormalities, and a lumbar puncture, which showed no relevant findings (Table 1).

Treatment with intravenous infusion of human immunoglobulin at a dose of 400 mg/kg/day was initiated for five days.

Neurology reevaluation (day 3)

After one day of treatment, the patient reported slight general improvement. On examination, flaccid quadriparesis persisted, with minimal preserved movement limited to the flexion and extension of the left second finger. Sensory findings revealed anesthesia and paresthesia in all four limbs, except the left second finger. Pinprick sensation was reduced in the right hemiface, with anesthesia on the left side, sparing the mandibular angle.

Systematic inconsistencies became apparent, not only in her clinical history and neurological examination but also in the documentation provided. Multiple written reports for the same MRI were noted, raising suspicions of forgery. Additionally, the MRI described by the patient was unusually written, reporting "multiple sclerosis lesions with gadolinium enhancement" but not describing the lesion signal changes or even their locations. These discrepancies prompted a request for a psychiatric evaluation, and the EMG with nerve conduction study was ultimately not performed.

Psychiatric consultation (day 3)

Further investigation uncovered critical aspects of her background. She disclosed having a nine-year-old daughter who lived with her parents. Her psychiatric history included an admission four years earlier for anxiety and sleep disturbances, attributed to a prior diagnosis of post-traumatic stress disorder (PTSD) from sexual abuse at the age of 14. She declined to provide further details about the abuse or family history, and there was no reported history of substance abuse. Treatment for PTSD included sertraline, titrated to a dose of 100 mg/day, which she discontinued after one year, and she denies having undergone any complementary therapies.

The patient claimed to be a junior resident doctor practicing at a nearby hospital and even provided her attending doctor's name but could not provide a Portuguese Medical Association ID number. After obtaining her consent, the hospital she mentioned was contacted and denied any record of her employment. Further discrepancies were noted in her reported timeline of medical school and residency training.

Her boyfriend, who had known her for less than a month, revealed that they had met in an online support group for healthcare providers where she had presented herself as a medical doctor. Additionally, she had restricted access to her medical records at the other hospitals by healthcare professionals.

During the observation, she appeared calm, cooperative, and oriented but exhibited growing restlessness and aggressive behavior when questioned. The patient was euthymic and demonstrated marked affective indifference, also referred to as belle indifference. Attention, speech, thought and perception without significant alterations, and her sleep and appetite were reported as regular. Importantly, no secondary gains, such as material benefits, advantages, or the avoidance of responsibilities (legal or otherwise), were identified. The patient exhibited multiple inconsistencies in the past history, along with clinical signs that collectively raised a strong suspicion of Munchausen syndrome.

Neurology reevaluation (day 3)

The patient was questioned regarding the inconsistencies encountered, to which she evaded responses, citing memory loss or the misplacement of test results. For example, the patient claimed to have two rare genetic conditions (TRAPS and DADA2) both diagnosed by genetic testing at the age of 13 in a private hospital. When asked for the results, the patient said that she could not provide them because "the hospital closed down years ago and never gave me the printed results." Of note, DADA2 was first described in 2014, while the patient claimed to have been diagnosed with this specific genetic disorder in 2004 [7].

On examination, her muscle tone was normal, with brisk reflexes (+++/+++) and bilateral flexor plantar response. Although she demonstrated absent voluntary limb or neck movement, involuntary muscle contractions were observed when she was distracted. Visual field testing revealed left homonymous hemianopsia; however, her blink reflex remained intact.

An event further substantiating the suspicion of MS occurred when the patient, who claimed to be unable to move her limbs, was observed texting her partner about our questions regarding the situation. Later that same day, she walked out of the hospital unaided and did not return, also missing the scheduled psychiatry appointment the following month.

The findings led to a final diagnosis of MS, characterized by an attempt to simulate an acute multifocal neurological syndrome. Treatment with immunoglobulin was discontinued, and psychiatric intervention was recommended. No further neurological investigations or treatments were deemed necessary. She was discharged with instructions for psychiatric and psychological follow-up, which she did not attend. According to the patient's boyfriend, she remained physically healthy and without any neurological symptoms during the following week, after which all contact with her was terminated.

## Discussion

Epidemiology

MS is rare, with most cases presenting dramatic clinical features in the emergency department simulating severe and serious illnesses. Unlike this case, MS is more commonly observed in young to middle-aged men who frequently change healthcare providers and simulate diverse medical issues, resulting in the lack of verifiable medical history [2]. Factitious disorders, however, are overall more common in women, with a female-to-male ratio of approximately 3:1 [8]. While physical examinations may highlight inconsistencies, this diagnosis of exclusion remains challenging due to the patients' ability to mimic symptoms convincingly. The prevalence of factitious disorders is estimated to be 0.2%-1% among hospital inpatients [2,9].

Etiology

The exact cause of MS remains unclear. However, psychosocial factors such as childhood trauma, abandonment, or the loss of a loved one are commonly reported [10,11]. Patients often fabricate illnesses to gain attention, affection, and a sense of belonging within the healthcare system. Their actions are not motivated by financial gain but rather by emotional needs, such as experiencing control over medical professionals or seeking the "rush" of medical procedures [9,10].

Psychiatric and trauma histories are prevalent in these patients, with depression and dynamic interpersonal conflicts frequently observed. In the current case, the patient's history of childhood sexual abuse, PTSD, and previous psychiatric admission for anxiety and sleep disturbances likely contributed significantly to the development of her condition. Her behavior, including fabricated professional credentials and inconsistencies in her medical history, may reflect a need for validation, attention, or control, potentially stemming from past trauma. Developmental influences, including abuse, grief, or enmeshment in family dynamics, may contribute to the disorder's complex origins [11,12]. Psychological theories propose three potential mechanisms, including intrinsic psychopathology such as mood, anxiety, or personality disorders; learned behavior reinforced by the attention or care received; and attachment deficits stemming from formative trauma. These mechanisms suggest that past and present stressors play a significant role in the development of factitious disorder [12].

Psychopathology

Comorbid psychiatric conditions are common, with depression being the most frequently reported [1,3]. This association may stem from shared risk factors such as childhood neglect, abuse, or traumatic life events. Alternatively, factitious disorder might emerge secondary to depression, serving as a form of self-harm or reflecting low self-esteem. While previous studies suggest a link between factitious disorder and suicide risk, the exact relationship remains unclear. Factors such as parental failures, substance abuse, and marital difficulties are also potential contributors to the disorder's manifestation [1,3,4].

Diagnosis

Patients with factitious disorder (FD), including MS, often present their symptoms dramatically, but their stories tend to be vague and inconsistent. They may exhibit a pattern of pathological lying (pseudologia fantastica) and possess extensive medical knowledge, sometimes even on par with healthcare professionals [13]. FD patients are drawn to conditions that prompt fast-track or protocol-driven admissions, such as chest pain, and may use substances such as insulin, anticoagulants, or thyroid hormones to induce illness [1,4].

Patients with FD tend to gravitate toward specialties managing complex medical conditions, such as cardiology or neurology, where it is harder to simulate illnesses due to advanced diagnostic tools. In neurology, factitious presentations often include seizure-like episodes, sensory deficits, or motor weakness, mimicking conditions such as epilepsy, stroke, or GBS. These neurological manifestations can be particularly challenging, underscoring the need for a careful clinical assessment and objective testing [6]. The increased attention and sympathy these specialties offer may be part of the reward motivating their actions [1,11].

Diagnostic Considerations

As per the DSM-5, MS is classified as a factitious disorder imposed on oneself. Diagnosis is based on atypical presentations that do not align with clinical findings, such as inconsistent laboratory results, scans, or physical examinations. The disorder is typically identified in adulthood and is often linked to a history of multiple unsuccessful treatments from various healthcare providers. Patients often exhibit unusual medical knowledge [3,5]. In this case, several inconsistent findings were noted. The clinical presentation had a sudden onset of symptoms, which is atypical for GBS, as it usually progresses gradually. Additionally, the presence of brisk reflexes in the lower limbs observed on the second day contradicted the initial findings, and there were variations in sensory responses between days 1 and 2. Absent radial and superficial abdominal reflexes suggested possible lower motor neuron involvement, while brisk lower limb reflexes indicated an upper motor neuron component, raising the possibility of mixed motor involvement or a functional disorder. The left-sided peripheral facial palsy was unusual for GBS, suggesting possible cranial nerve involvement or an alternative diagnosis [6]. Complementary examinations, including cerebrospinal fluid (CSF) analysis and cranial and cervical CT, were normal, challenging the diagnosis of GBS. These findings highlight the importance of a thorough neurological evaluation to clarify the diagnosis and identify any inconsistencies.

The diagnostic process involves first ruling out common medical conditions and considering the possibility of less common organic diseases, such as mitochondrial disorders. It is also essential to differentiate FD from somatoform disorders (where symptoms are not intentionally fabricated) and malingering (where symptoms are faked for personal gain). Key signs of FD include a dramatic but inconsistent medical history, worsening symptoms after treatment begins, and a reluctance to involve family members or past healthcare providers in the patient's care. In severe cases, patients may fabricate their personal history and actively seek treatments, tests, or surgeries despite potential harm [8].

Red Flags and Risk Factors for MS

Certain clinical red flags and atypical behaviors may raise suspicion for MS, such as patients presenting with inconsistent or dramatic symptoms, often without medical confirmation. A detailed history and physical examination are crucial for addressing the immediate symptoms and for considering MS as a potential diagnosis [3,14].

Patients may sometimes leave against medical advice and seek care at another hospital. Identified risk factors include being a woman, being unmarried, and working in healthcare. Individuals with borderline or histrionic personality traits, as well as those with a history of sexual abuse, are also at higher risk [7,12-15].

Differential Diagnosis of MS

Munchausen syndrome must be differentiated from other psychiatric disorders, including conversion disorder, hypochondriasis, malingering, somatization disorders, and MS by proxy. Unlike malingering, which is motivated by external gains such as financial benefits, MS is driven by an intrinsic desire for attention or care. Conversion disorder, while also presenting with physical symptoms, arises from unconscious psychological stress, whereas MS involves the deliberate fabrication or exaggeration of symptoms for personal attention [14-16]. Similarly, self-mutilation serves a distinct purpose compared to MS, as it is typically employed to alleviate emotional tension rather than to seek attention or affection [16].

Treatment and prognosis

Effective treatment for MS remains elusive, and many patients refuse psychiatric help, often leaving the hospital before a diagnosis is made. Approaches such as confronting the patient early and offering long-term psychotherapy have been suggested, though results are mixed. Medication, including antidepressants and antipsychotics, may be considered, but their efficacy in managing MS is uncertain [17].

Psychiatric Intervention

Psychiatric intervention is essential for diagnosis, managing comorbidities, and providing long-term care. The goal is to reduce harmful medical procedures, maintain the doctor-patient relationship, and focus on psychiatric care [11,15].

Multidisciplinary Care

MS patients often have poor treatment adherence, and improvements are rare. Most treatments are conducted in hospital settings for a short time, which limits long-term outcomes. Treatment must involve psychiatry, focusing on understanding the patient's emotional needs, exploring motivations, and providing long-term support [3,4].

Long-Term Management

This typically includes developing healthier coping mechanisms, taking responsibility for recovery, and addressing underlying psychiatric conditions, such as depression. Psychiatric comorbidities must be treated for a better prognosis, as some studies suggest that FD may improve with age [11].

Poor prognostic factors are often associated with the chronicity of symptoms, the severity of adopted behaviors (given the risk of medical and psychological complications), nonadherence to treatment, the lack of insight, psychiatric comorbidities (personality disorders and mood disorders), a history of unresolved trauma, and limited social and/or familial support [1].

Recovery from MS is extremely rare, with a small number of patients acknowledging their self-induced illness and consenting to psychiatric treatment [14]. The disorder is often chronic, and while manifestations may temporarily improve, relapses are common. Some patients may improve as they age, particularly with the presence of treatable psychiatric conditions [14,15].

## Conclusions

MS is a complex and challenging psychiatric disorder characterized by the intentional fabrication of symptoms to obtain medical attention, often leading to unnecessary medical procedures, prolonged hospitalizations, and increased healthcare costs. Its rarity and underdiagnosis and the lack of systematic research contribute to the absence of reliable diagnostic criteria and effective treatment protocols. Patients frequently deny their diagnosis, refuse treatment, and seek care from multiple providers, complicating clinical management and research efforts. An interdisciplinary approach, including psychiatric support, careful monitoring, and collaboration among healthcare providers, is essential to mitigate the risks and improve outcomes. While prognosis is generally poor and recovery is rare, long-term psychiatric care, behavioral therapy, and addressing underlying emotional issues may offer some benefit, underscoring the need for further research and awareness within the medical community.
